# Determination of respiratory syncytial virus epidemic seasons by using 95% confidence interval of positivity rates, 2011–2021, Germany

**DOI:** 10.1111/irv.12996

**Published:** 2022-04-29

**Authors:** Wei Cai, Ralf Dürrwald, Barbara Biere, Brunhilde Schweiger, Walter Haas, Thorsten Wolff, Silke Buda, Janine Reiche

**Affiliations:** ^1^ Unit 36, Respiratory Infections, Department of Infectious Disease Epidemiology Robert Koch Institute Berlin Germany; ^2^ Unit 17, Influenza and Other Respiratory Viruses, Department of Infectious Diseases, National Influenza Centre Robert Koch Institute Berlin Germany; ^3^ Unit 17, Influenza and Other Respiratory Viruses, Department of Infectious Diseases, Consultant Laboratory for RSV, PIV and HMPV Robert Koch Institute Berlin Germany

**Keywords:** acute respiratory infection, confidence interval, epidemic season, respiratory syncytial virus, surveillance

## Abstract

Based on our national outpatient sentinel surveillance, we have developed a novel approach to determine respiratory syncytial virus (RSV) epidemic seasons in Germany by using RSV positivity rate and its lower limit of 95% confidence interval. This method was evaluated retrospectively on nine RSV seasons, and it is also well‐suited to describe off‐season circulation of RSV in near real time as observed for seasons 2020/21 and 2021/22 during the COVID‐19 pandemic. Prospective application is of crucial importance to enable timely actions for health service delivery and prevention.

## 1 INTRODUCTION

Respiratory syncytial virus (RSV) is the major cause of acute lower respiratory tract infection (ALRI) in children.^1^ Globally, 33.1 million episodes of ALRI were caused by RSV infection which resulted in estimated 3.2 million hospital admissions, and 59,600 in‐hospital deaths in children younger than 5 years in 2015.^2^ To date, no approved vaccine against RSV exists.^3^ In order to prevent severe ALRI due to RSV, passive immunoprophylaxis with palivizumab can be carried out in infants at high risk.^4^

RSV causes seasonal epidemics worldwide.^5^ In Europe, an average RSV season starts in the beginning of December, peaks in early February, and continues until early April.^6^ So far, there is no accepted method for defining start and end of RSV epidemic seasons, although few methods have been recently recommended.^7^

In Germany, RSV activities were commonly investigated within our national outpatient sentinel surveillance, and RSV epidemic seasons have been determined retrospectively.^8^, ^9^ This study aimed to establish a prospective, sensitive, and sustainable method to determine the start and end of RSV epidemic seasons on population level.

## 2 RSV DETECTIONS

Specimens (nasal or throat swabs, and nasopharyngeal swabs) from outpatients of five age groups (0–4, 5–14, 15–34, 35–59, and >60 years old) with acute respiratory infection (ARI) were collected year‐round by physicians participating in the national sentinel surveillance and sent to the Robert Koch Institute (RKI, Berlin, Germany) where they were prospectively tested for RSV by real‐time reverse transcription PCR.^10^

**FIGURE 1:**
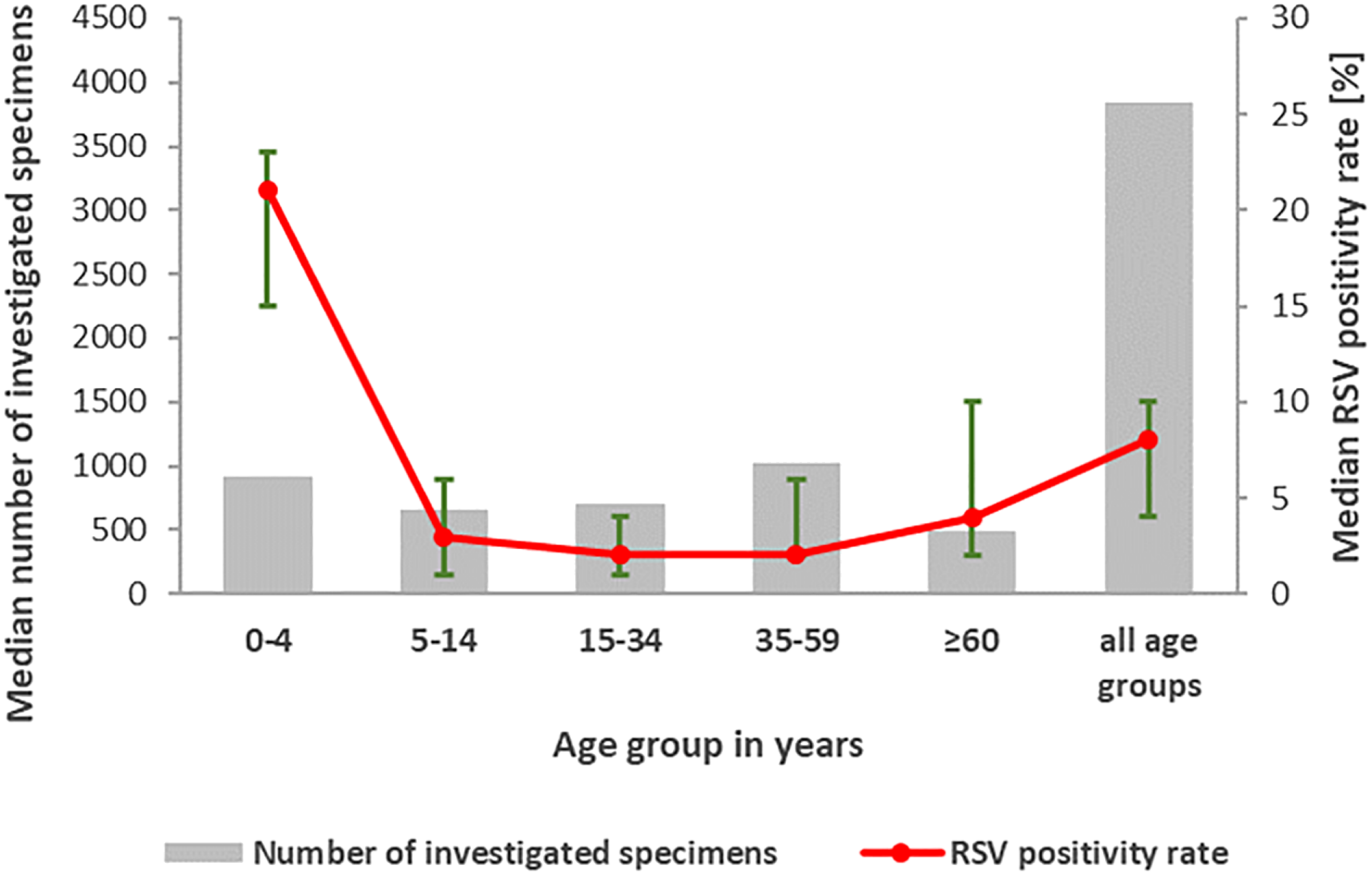
Median number of investigated specimens (gray bars) and median RSV positivity rate (red line) with range (green error indicators) in different age groups, calendar weeks 40 to 20 in seasons 2011/12 to 2019/20

From 2011/12 to 2019/20, a total of 33,351 RSV positives were detected within calendar weeks 40 to 20, the time of regular RSV activity in Germany. Although RSV detections were observed in all age groups, median positivity rates (PR) were highest among young children aged 0 to 4 years (Figure 1). Further, median RSV PR in young children were 2.6‐fold higher compared with median PR in all ages (nonparametric Mann–Whitney *U* test, *P* = .0003, Stata® version 17). Because robust and reliable RSV surveillance data are of crucial importance, RSV PR among 0 to 4 years old children was selected for the determination of RSV epidemic season solely.

## 3 DETERMINATION OF RSV SEASONALITY

**FIGURE 2:**
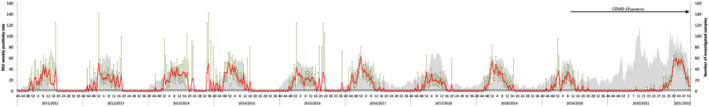
RSV weekly positivity rates (red) with 95% confidence interval (green error bars) for age group 0–4 years by calendar week from season 2011/12 to 2021/22. A season was defined from calendar week 40 up to week 39 of the following year. Gray shadows represent number of investigated samples with the corresponding scale on secondary axis. The black line marks the threshold (5%, lower limit of 95% confidence interval of the RSV positivity rate). Black arrow displays the course of COVID‐19 pandemic

Among children aged 0 to 4 years, the number of RSV positive and negative tested cases were sorted by season and calendar week (collection date, if not available then received date). For each calendar week, the PR and the associated 95% confidence interval (95% CI) were calculated with Stata® version 17. Both RSV PR and the corresponding 95% CI were plotted against the calendar week of the seasons 2011/12 to 2021/22 (Figure 2). A high PR can be associated with a low number of investigated (received) samples and single RSV positive detections and will moreover result in a less precise estimate of the 95% CI with probably reduced lower limit of 95% CI (Figure 2). To circumvent such unlikely high RSV PRs, the lower limit of the 95% CI was used herein to determine the RSV epidemic season. Within an iterative process, manually different thresholds were thoroughly evaluated to determine the cut off for the start and end of retrospective RSV epidemic seasons. Then, the start of the RSV epidemic season was defined as the first of two consecutive weeks in which the lower limit of 95% CI of the RSV PR exceeds 5% among 0 to 4 years old children. The RSV epidemic season ends by the week that precedes first of two consecutive weeks, in which the lower limit of 95% CI of the RSV PR drops below 5%. One gap week below the threshold was allowed.

**FIGURE 3:**
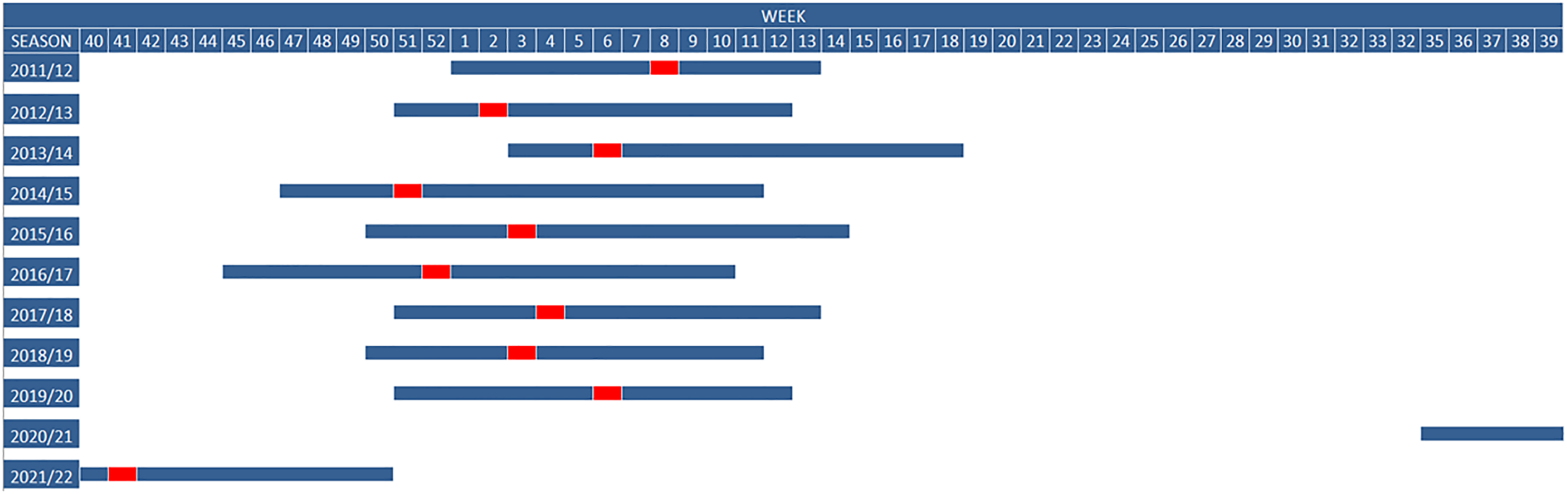
Timing of RSV epidemic seasons (blue) by calendar week from 2011 to 2021 in Germany. The peak week is colored in red

Among 0 to 4 years old children, an RSV epidemic season could be retrospectively assigned to each season from 2011/12 to 2019/20 (Figures 2 and 3). The median start of RSV epidemic seasons was in calendar week 50 (range: 45–3), peaked in median in calendar week 8 (range: 51–10), and ended in median in week 12 (range: 10–18). The median length of the RSV season was 15 weeks (range: 13–18).

In contrast, during the COVID‐19 pandemic starting in calendar week 11 in 2019/20,^11^ there was almost no RSV detected within the time of RSV epidemics as observed for the seasons 2011/12 to 2019/20 (median weeks 50–12) among 0 to 4 years old children. Between calendar weeks 9 and 30 in 2020/21, only sporadic RSV cases occurred. The number of RSV cases and the corresponding RSV PR increased in calendar week 31. According to our definition of the timing of an RSV epidemic season, in 2020/21, the RSV season began in calendar week 35 (PR: 13%, 95% CI: 5.2–24.1) and continued until calendar week 50 (PR: 26%, 95% CI: 15.3–39.0) in season 2021/22 (Figures 2 and 3).

## 4 ETHICAL STATEMENT

The German national ARI sentinel surveillance has been approved by the Charité‐Universitätsmedizin in Berlin Ethical Board (Reference EA2/126/11).

## 5 DISCUSSION

Using German national ARI sentinel surveillance data of 0–4 aged outpatients, we have developed a novel approach to describe RSV epidemic seasons both retrospectively and prospectively. Although sentinel practitioners were encouraged to continuously collect specimens year‐round, the number of specimens send to RKI was considerably low between calendar weeks 21 and 39 for seasons 2011/12–2018/19. A low sample size will result in a high RSV PR, which is most likely far away from the “true value.”^12^ The width of the 95% CI increases, and the lower limit of 95% CI decreases correspondingly as the sample size decreases. Thus, the lower limit of 95% CI of the RSV PR was used herein for the determination of RSV epidemic season. This is similar to the determination of the influenza seasonality in Germany where the cutoff was set 10% as the lower limit of 95% CI of PR.^13^

Based on this 95% CI method, RSV epidemic seasons 2011/12 to 2019/20 started in median in calendar week 50 (range: 45–3) and ended in median in week 12 (range: 10–18) in Germany. Calculations for the determination of RSV epidemic seasons on the same setting applying the 1.2% method defined a similar start (week 48) or end (week 15) as determined by our novel approach.^6^, ^9^ In addition, the median length of the RSV epidemic season in Germany was 15 weeks (range: 13–18) as determined in this study, and 17 (11–22) weeks as calculated with the retrospective 1.2% threshold.^6^ The median length of RSV epidemic seasons based on sentinel surveillance systems in the European Union/European Economic Area was 16 (range: 9–24) weeks.^6^

For a further comparison, we analyzed our sentinel data with a threshold of a weekly percentage of 3%,^13^ which resulted in a comparative earlier start (week 47, range: 45–50) and longer duration (length 22 weeks, range: 20–26) of RSV epidemic season. This might be most likely caused by low sample numbers at the start and end of the RSV season. For the same reason, we could not apply the 10‐fold baseline method, a near real‐time approach, to our setting, because no threshold could be determined.^13^ Further, we examined our data set by the moving epidemic method, which has been recently validated for characterization of RSV epidemics in the Netherlands.^14^ Based on optimum slope parameter of 2.2, the obtained average epidemic start week of 51 and average week length of 14 confirmed calculations by the 95% CI method.

As for many other European countries, in the winter season 2020/2021, there were almost no RSV cases detected in Germany.^15^ Reasons might be seen in the COVID‐19 pandemic and associated non‐pharmaceutical interventions (NPI) strategies, which suppressed transmission not only of SARS‐CoV‐2 but also of other respiratory viruses including RSV.^10^ Efforts in vaccination campaigns against SARS‐CoV‐2 led to loosening of NPI interventions in many fields since May 2021, thus leading to an increase of RSV starting late in calendar week 35 in season 2020/21. Group of 0 to 4 years old children was most affected in season 2020/21 and 2021/22, respectively, thus confirming convincingly that this age group provides reliable data for determination of RSV epidemic seasons in our sentinel surveillance.

## 6 CONCLUSIONS

We have developed a method that defines the start and end of an RSV epidemic season. The 95% CI method is reliable working on consistent sentinel data as well as in systems with a small weekly sample size. On a basis of timely receipt and testing of specimens for RSV, this method can be used in near real time, which had been proven in this unusual RSV off‐season circulation in seasons 2020/21 and 2021/22 during the COVID‐19 pandemic. Prospective use enables necessary flexible and timely actions in RSV prevention and control on population level.

## AUTHOR CONTRIBUTIONS

**Wei Cai:** Conceptualization; data curation; formal analysis; investigation; methodology; project administration; validation; visualization. **Ralf Dürrwald:** Conceptualization; investigation; resources; supervision. **Barbara Biere:** Data curation; investigation; methodology. **Brunhilde Schweiger:** Conceptualization; investigation; resources; supervision. **Walter Haas:** Resources; supervision. **Thorsten Wolff:** Conceptualization; resources; supervision. **Silke Buda:** Conceptualization; investigation; methodology; project administration; resources; supervision; validation. **Janine Reiche:** Conceptualization; data curation; formal analysis; investigation; methodology; project administration; resources; supervision; validation; visualization.
